# Endogenous Endophthalmitis From Methicillin-Resistant Staphylococcus aureus Bacteremia Treated With Ceftaroline

**DOI:** 10.7759/cureus.22216

**Published:** 2022-02-14

**Authors:** Daniel Ho, Nicola A Clayton, Bruce Silverstein, Alan Koff

**Affiliations:** 1 Internal Medicine, University of California Davis Medical Center, Sacramento, USA; 2 Pharmacy, University of California Davis Medical Center, Sacramento, USA; 3 Ophthalmology, Shasta Eye Medical Group, Redding, USA; 4 Infectious Diseases, University of California Davis Medical Center, Sacramento, USA

**Keywords:** methicillin resistant staphylococcus aureus (mrsa), ceftaroline, vancomycin failure, bacteremia, endogenous endophthalmitis

## Abstract

Methicillin-resistant *Staphylococcus aureus* (MRSA) bacteremia may lead to endogenous endophthalmitis (EE), an uncommon disease process described mainly in case series and case reports. It is considered an ophthalmologic emergency requiring prompt diagnosis and treatment with systemic intravenous and intravitreal antibiotics to improve mortality and prevent blindness. The management of MRSA EE represents a challenge, as there are no established guidelines for treatment or outcomes. In our case report, we demonstrate that ceftaroline may be considered as an alternative option to treat MRSA EE in lieu of vancomycin due to drug-induced rash, given the promising penetration of the blood brain barrier and low minimum inhibitory concentration (MIC) values for most *Staphylococcus aureus* isolates.

## Introduction

Methicillin-resistant *Staphylococcus aureus* (MRSA) bacteremia causes significant morbidity and mortality due to its propensity for endothelial dysfunction and tissue invasion, which potentially can lead to metastatic infection to any site of the body [1]. Rarely, intraocular seeding may occur resulting in endogenous endophthalmitis (EE), associated with a high risk of blindness if not promptly treated [2]. To preserve vision, patients require sufficient intravitreal antimicrobial drug levels as well as systemic intravenous therapies preferentially utilizing agents that penetrate the blood-ocular barrier. These constraints limit potential therapeutic options. Ceftaroline is a fifth-generation cephalosporin beta-lactam with activity against MRSA and is Food and Drug Administration (FDA)-approved for community-acquired pneumonia and skin soft-tissue infections [3]. Due to its broad-spectrum activity and good tolerability, ceftaroline has been used off-label as salvage therapy for bacteremia, bone/joint infections, and central nervous system infections [3]. The use of ceftaroline for MRSA EE is uncommon but represents a promising therapeutic option given treatment in our case report in the setting of a vancomycin-related drug rash.

## Case presentation

An elderly female, with a past medical history of dementia, congestive heart failure, atrial fibrillation, diabetes mellitus, hypertension, and hypothyroidism, presented to the hospital with two days of confusion and fever. 

On admission, her temperature was 103ºF. The cardiovascular exam revealed an irregular rhythm, lungs were clear to auscultation, and the abdominal exam was unremarkable. On skin exam, she had a sacral ulcer with purulent discharge. Labs were notable for a white blood cell count of 11.6 x 103/mm^3^ with a normal differential, hemoglobin of 15.6 g/dL, and platelets of 601 x 103/mm^3^. Her chemistry panel was unremarkable. Hemoglobin A1c was 10.5%. Sacral wound and peripheral blood cultures were positive for MRSA.

Three days into her course, she developed severe right eye pain, blurry vision, photophobia, watery discharge, and headache. Her right eye exam was notable for conjunctival injection, chemosis, and pain with extraocular movements. Slit-lamp exam revealed cellular debris and a 1 mm hypopyon in the anterior chamber. There were significant synechiae with superotemporal sparing of the right pupil. Ocular ultrasound confirmed right vitreous debris while tonometry revealed normal intraocular pressures. The constellation of MRSA bacteremia and the development of acute ocular symptoms were consistent with MRSA EE.

In addition, magnetic resonance imaging of the brain disclosed three foci of restricted diffusion within the right periatrial and periventricular white matter as well as the right parietal cortex, findings that could represent acute infarct from septic emboli. No vegetations were visualized on the transthoracic echocardiogram. Repeat blood cultures were negative. A transesophageal echocardiogram was deferred given the rapid clearance of blood cultures, development of in-hospital delirium, risks associated with sedation, and intent to presumptively treat for at least six weeks.

One-time intravitreal injections of vancomycin 1 mg/0.1 mL and ceftazidime 2 mg/0.1 mL were administered into the mid-vitreous chamber. In addition, the patient received topical ophthalmic atropine, prednisolone, and moxifloxacin. Due to improvement in symptoms with antimicrobial therapy, ophthalmology determined that a vitrectomy was not indicated. 

**Figure 1:**
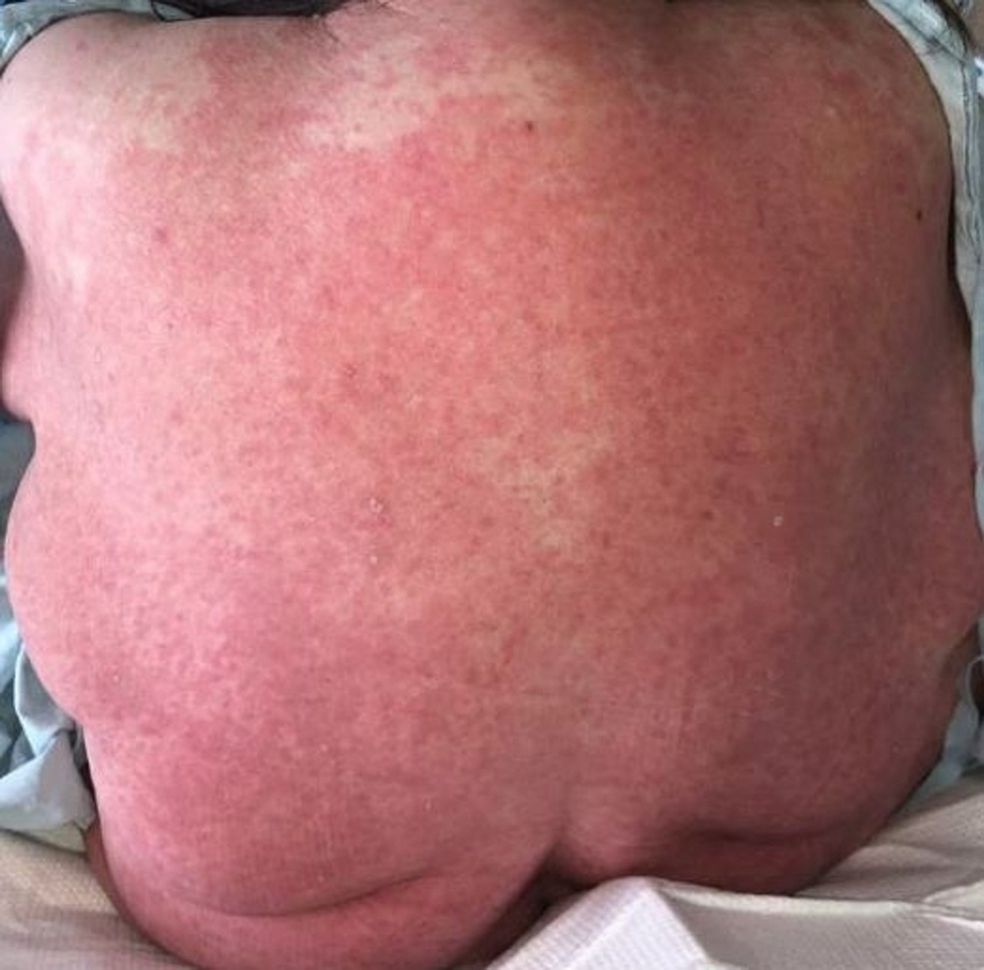
A diffuse morbilliform exanthem attributed to vancomycin in our patient

On hospital discharge, a peripherally inserted central catheter was placed with a plan for six weeks of intravenous vancomycin. Less than two weeks into her treatment course, she developed a morbilliform drug rash attributed to vancomycin, shown in Figure 1. Thus, alternative agents were considered.

Intravenous daptomycin appears to have limited penetration of the blood-retinal barrier (BRB) at standard dosing and may not result in high enough drug concentrations to treat EE [4]. Linezolid and tedizolid were felt to pose too high a risk for optic neuritis in a patient who was unable to reliably notify of new vision changes due to underlying dementia. There are no data on ceftaroline penetration of the blood-retinal barrier but two animal model studies and one human case study suggest a cerebrospinal fluid (CSF) penetration of 14-15% through inflamed meninges and 3% across uninflamed meninges [5-7]. Given that the BRB has been shown to be more permeable than the blood-brain barrier and given an MIC50 and MIC90 of 1 mg/dL for community-acquired MRSA and hospital-acquired MRSA isolates in the United States, she was transitioned to ceftaroline 600 mg every eight hours [8].

At her six-week follow-up, due to mild residual ocular inflammation reported by her ophthalmologist, ceftaroline was extended to eight weeks and then discontinued. Two months following the cessation of antibiotic therapy, she demonstrated no further evidence of active inflammation though she had residual right eye scarring and visual acuity remained poor at 20/400.

## Discussion

Endophthalmitis is an intraocular infection considered an ophthalmologic emergency due to the threat of vision loss, which is termed exogenous if caused by the external entry of pathogens (from surgery or trauma) or endogenous if caused by hematogenous seeding typically from bacteria or fungi invading across the blood-ocular barrier [2]. EE is rare, as it accounts for 5-10% of all cases of endophthalmitis, with medical literature currently comprising mainly of case reports and case series; the consequence of this being no standardized treatment guidelines [2].

The diagnosis of EE should be suspected in patients with bacteremia or fungemia with the development of acute ocular symptoms such as vision changes, eye pain, or photophobia [9]. Hypopyon may be seen as well, consistent with inflammation in the anterior chamber. The majority of patients who develop EE are those with underlying risk factors, such as diabetes mellitus, renal failure, indwelling catheters, or immunocompromised states such as with human immunodeficiency virus [2,9].

MRSA EE is very rare and is associated with high mortality and poor visual outcomes. Prompt diagnosis is critical in prognosis, as Ho et al. demonstrated that MRSA EE had high rates of retinal detachment when treatment with systemic antibiotics and/or intravitreal injections were delayed for over two weeks and that rates of enucleation could be as high as 47.1%, likely due to delay in diagnosis from the late-onset presentation [10]. Despite adequate therapy, patients may still have poor outcomes, as Ness et al. described three cases of patients with MRSA EE and despite treatment, two of the three became blind, one of which required enucleation, and the third had some visual recovery though this was limited [11]. A retrospective study by Nishida et al. proposed that the visual prognosis of EE may depend on the patient’s initial visual acuity as well the virulence of the organism and host conditions [12].

The treatment of EE consists of systemic antibiotics to treat the bacteremia and locally targeted therapy with intravitreal antibiotics or vitrectomy [9,12]. Yonekawa et al. recognized the lack of established treatment guidelines and proposed that systemic antibiotics supplemented with intravitreal antibiotics without routine vitrectomy may yield favorable outcomes [13]. In severe cases, surgical vitrectomy may be recommended to decrease the burden of infection [13]. Vancomycin is the recommended antibiotic for MRSA EE, as 32 isolates of *Staphylococcus aureus* obtained from intraocular specimens had 100% susceptibility to vancomycin [14]. The antibiotic sensitivities of gram-positive organisms in the nine cases from Yonekawa et al. had 100% sensitivity to vancomycin as well [13]. However, the relatively poor penetration of vancomycin through the BRB, the narrow therapeutic range of vancomycin, and the MIC50 and MIC90 of 1 mg/dL for *Staphylococcus aureus* isolates may lead to the increased rates of treatment failure associated with MRSA EE [8].

Our patient was initially on first-line therapy with intravenous vancomycin, however, she was transitioned to ceftaroline due to vancomycin-related drug rash. Ceftaroline is increasingly becoming used as off-label salvage therapy for MRSA bacteremia though it is uncommonly reported for use in MRSA EE. Currently, ceftaroline is FDA-approved for community-acquired pneumonia and skin soft-tissue infections caused by *Staph. aureus* [3]. In a multicenter study, off-label use of ceftaroline as salvage therapy for MRSA bacteremia had clinical success up to 70% [15]. A retrospective study by Polenakovik et al. found clinical success of 74.2% using ceftaroline to treat MRSA bacteremia with or without concomitant daptomycin as alternative therapy when standard therapies failed [16]. Ho et al. found that six patients undergoing salvage monotherapy with ceftaroline for MRSA bacteremia or endocarditis had a rapid clearance of bacteremia, with a clinical success rate of 83.3% [17]. These studies suggest that off-label ceftaroline is a promising treatment for MRSA bacteremia in the setting of vancomycin failure. Moreover, as seen in our case report, ceftaroline may be a well-tolerated and effective alternative option to treat MRSA EE as well, though further investigation is needed. For our patient, upon completion of her course of intravenous ceftaroline, she demonstrated resolution of her active infection though visual acuity remained poor, as expected given the poor prognosis of the disease process.

## Conclusions

MRSA endogenous endophthalmitis is a rare entity as described mainly in case series and case reports. It is an ophthalmologic emergency and should be suspected in patients with bacteremia with the development of acute ocular symptoms. Prompt treatment with both systemic intravenous and intravitreous antimicrobial therapy, with or without vitrectomy, may improve outcomes. Vancomycin is the first-line systemic intravenous antibiotic of choice. Ceftaroline may be considered as an alternative treatment, when unable to use vancomycin, given its penetration through the blood-brain barrier and generally low community minimum inhibitory concentrations (MICs) among MRSA isolates. Due to the high morbidity and mortality associated with bacteremia and its complications, MRSA EE may require further large-scale studies to establish more definitive guidelines for treatment and outcomes.
